# Seroprevalence Trends of Antibodies to SARS‐CoV‐2 in South Korea, 2021–2022: A Repeated Cross‐Sectional Study

**DOI:** 10.1111/irv.70117

**Published:** 2025-06-03

**Authors:** Ah‐Ra Kim, Chiara Achangwa, Hyeon Nam Do, Eun Young Jang, Yukyung Nam, Seonghui Cho, Taegu Kim, Hye‐Sook Jeong, Gi‐eun Rhie, Kyungwon Oh, Seunghyun Lewis Kwon, Seunghyeon Lee, Junewoo Lee, Sukhyun Ryu

**Affiliations:** ^1^ Division of Clinical Research for Vaccine, Center for Vaccine Research, National Institute of Infectious Diseases National Institute of Health Cheongju South Korea; ^2^ Department of Preventive Medicine, College of Medicine The Catholic University of Korea Seoul South Korea; ^3^ Division of Vaccine Development, Center for Vaccine Research, National Institute of Infectious Diseases National Institute of Health Cheongju South Korea; ^4^ Department of Statistics Korea University Seoul South Korea; ^5^ Center for Vaccine Research National Institute of Infectious Diseases Cheongju South Korea; ^6^ Division of Health and Nutrition Survey and Analysis, Department of Chronic Diseases Prevention and Control Korea Disease Control and Prevention Agency Cheongju South Korea; ^7^ Division of Immunization Services, Department of Healthcare Safety and Immunization Korea Disease Control and Prevention Agency Cheongju South Korea

**Keywords:** antibody, community cohort, disparity, SARS‐CoV‐2, sero‐surveillance, surveillance

## Abstract

**Background:**

Monitoring immunity levels nationwide and identifying disparities are important to prepare for future pandemics. However, data regarding changes in the seroprevalence of SARS‐CoV‐2 and disparities in consecutive epidemic waves using existing surveillance systems are limited.

**Methods:**

We conducted a cross‐sectional serosurvey using 11,506 residual serum samples collected from the Korean National Health and Nutrition Examination Survey between January 2021 and December 2022. Antibodies to the SARS‐CoV‐2 spike (anti‐S) protein, indicative of vaccination or past infection of SARS‐CoV‐2, and nucleocapsid (anti‐N) protein, indicating infection, were quantified. Then, we applied post‐stratification weighting through the bootstrap resampling based on the age and sex distribution of the South Korean population. We used regression models to identify any disparities in the seropositivity prevalence ratio (PR) across different epidemic waves of SARS‐CoV‐2 and demographics in the population.

**Results:**

We identified that the anti‐S seropositivity gradually increased after the COVID‐19 vaccination rollout, whereas anti‐N seropositivity increased after the SARS‐CoV‐2 Omicron variant was introduced in Korea. Anti‐S seropositivity PR was 0.12–0.76 times lower in individuals < 18 years than in elderly individuals ≥ 65 years during Waves 4–5 (July 2021 to June 2022). Anti‐N seropositivity PR was 1.25–1.83 times higher in individuals less than 64 years than in elderly individuals during Waves 5–7 (January 2022 to December 2022).

**Conclusions:**

Our findings provide insights into the dynamic changes in immunity levels among the Korean population after the COVID‐19 vaccine rollout and the introduction of the Omicron variant. Identifying the disparity in seroprevalence in the study population during the pandemic by using the existing surveillance system provides helpful information to develop future pandemic preparedness plans for the population.

## 1 Introduction

Severe acute respiratory syndrome coronavirus 2 (SARS‐CoV‐2) was first identified in South Korea on January 20, 2020, and the initial epidemic waves of coronavirus disease 2019 (COVID‐19) were observed during the spring of 2020 [1]. South Korea used a very successful SARS‐CoV‐2 infection control strategy including isolation of confirmed cases, contact tracing, extensive testing, and timely quarantine of all contacts throughout the COVID‐19 pandemic during 2020–2021 [2, 3]. In February 2021, in South Korea, the adenovirus vector‐based vaccine (ChAdOx1 nCoV‐19) against SARS‐CoV‐2 was initially authorized for adults, and in March 2021, the mRNA vaccine (BNT162b2) was introduced for children aged > 5 years [4, 5]. In December 2021, the first dose of the COVID‐19 vaccination rate reached 81% of the overall population in South Korea [6], and Korea began to relax the public health and social measures in mid‐January 2022 when the early phase of the Omicron variant was introduced [7]. Substantial public health resources were put in place for monitoring the number of SARS‐CoV‐2 infections during 2020–2021, but a surge in the number of asymptomatic SARS‐CoV‐2 infections among vaccinated individuals limited the efforts to quantify the exact burden of SARS‐CoV‐2 infections in Korea [8].

Seroprevalence studies have played a critical role in understanding the dynamics of SARS‐CoV‐2 transmission and population‐level susceptibility. Although there are global seroprevalence studies for SARS‐CoV‐2 to assess the impact of natural infection and vaccination on population immunity [9, 10, 11, 12], Korea presented a distinct case, which was characterized by rigorous contact tracing with testing and targeted interventions, and delayed large‐scale community transmission until the COVID‐19 vaccine rollout [2]. This unique epidemiological trajectory allowed us to investigate the vaccine‐induced and infection‐induced immunity in a population that largely avoided the early COVID‐19 pandemic in South Korea. By examining the temporal trends in seroprevalence across different demographic and geographic groups in South Korea, our study seeks to fill an important gap in the global understanding of immune dynamics during the COVID‐19 pandemic. Here, we report the results of the community‐based cross‐sectional serosurveys of anti‐S and anti‐N antibodies from the beginning of January 2021 through December 2022 in Korea.

## 2 Methods

### 2.1 Study Design

We conducted a cross‐sectional serosurvey through the Korean National Health and Nutrition Examination Survey (KNHANES). KNHANES is a national surveillance system that has been used to assess the health and nutritional status of Koreans since 1998. The target population of KNHANES comprises non‐institutionalized Korean individuals residing in community settings, excluding those in long‐term care facilities, nursing homes, correctional institutions, and other residential care settings. We analyzed all serum samples collected from the KNHANES. The sampling design of KHANES for the population follows a multi‐stage clustered probability design from 200,000 geographically defined primary sampling units (PSU) across Korea [13]. Each PSU was selected based on national census data to ensure comprehensive geographic coverage. Then, a subset of households within each PSU is randomly selected to minimize selection bias.

To ensure that the analyzed subset remained demographically representative (i.e., age, sex, and geographic region), we compared the distribution of these demographic factors between the participants from the KNHANES sample and those of the general population in Korea. To further account for any residual demographic imbalances, we applied post‐stratification weighting through the bootstrap resampling based on the age and sex distribution of the South Korean population, using census data as a reference [14]. Furthermore, we conducted multivariable regression analyses to adjust for the sensitivity and specificity of the laboratory test [5], and other potential confounding factors in seropositivity trends across demographic groups [15, 16].

In this study, residual serum samples collected between January 20, 2021, and December 22, 2022, from participants enrolled in the KNHANES cohort were analyzed. Data collected included information on age, sex, residential region, and date of specimen collection. Data including history of previous SARS‐CoV‐2 infection and COVID‐19 vaccination status were extracted from the Disease Management Integrated Information System and Immunization Registry Information System of the Korea Disease Control and Prevention Agency (KDCA) to avoid recall bias from the participants [17]. This study was approved by the institutional review board of the KDCA (IRB No. 2018‐01‐03‐3C‐A). Informed consent was obtained from all study participants or their legal guardians, as appropriate.

### 2.2 Laboratory Analysis of Serum Specimens

All serum specimens collected were tested for SARS‐CoV‐2 antibodies at the Korean National Institute of Infectious Diseases. The anti‐SARS‐CoV‐2 antibodies against the spike protein and the nucleocapsid protein were detected using two commercially available anti‐SARS‐CoV‐2 immunoglobulin immunoassays. The Elecsys® Anti‐SARS‐CoV‐2 N immunoassay (Roche Diagnostics International Ltd., Rotkreuz, Switzerland) was used to detect antibodies against the SARS‐CoV‐2 nucleocapsid protein (anti‐N) known to develop during a natural infection only [5, 18]. Antibodies against the SARS‐CoV‐2 spike protein (anti‐S), which are known to develop from both natural SARS‐CoV‐2 infection and COVID‐19 vaccination, were measured using the Elecsys Anti‐SARS‐CoV‐2 S immunoassay (Roche Diagnostics International Ltd.) [16, 19]. All tests were performed according to the manufacturer's instructions.

### 2.3 Statistical Analysis

**FIGURE 1:**
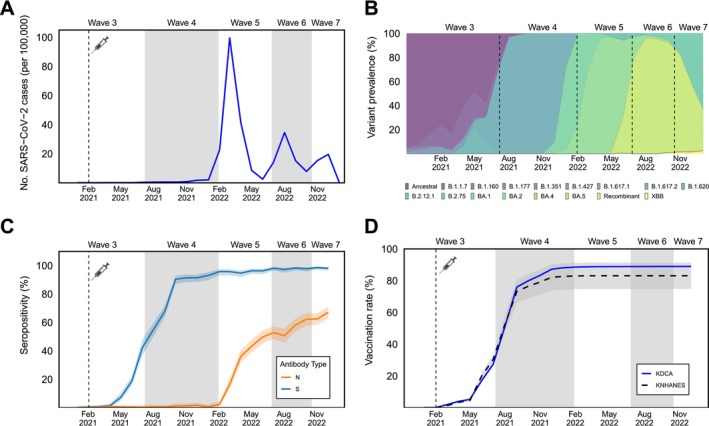
Temporal changes in SARS‐CoV‐2 epidemiology and antibody seropositivity in South Korea, 2021–2022. Changes in SARS‐CoV‐2 epidemiology including (A) number of infections and (B) percent prevalence of SARS‐CoV‐2 variant reported by the Korea Disease Control and Prevention Agency (KDCA). (C) anti‐S and anti‐N seropositivity by the current study (Korean National Health and Nutrition Examination Survey, KHANES). The orange colored line represents the monthly anti‐S antibody seropositivity, and the blue colored line represents the monthly anti‐N antibody seropositivity. The grey area surrounding the lines represents the corresponding 95% confidence intervals. (D) Monthly accumulated vaccination rate comparison between nationwide data (KDCA, blue solid line) and the current study (KHANES, black dashed line). The area surrounding the lines represents the corresponding 90% confidence intervals of the vaccination rate of the current study.

We categorized samples by time into epidemiological waves that corresponded to the Korean national classification of epidemic waves, defined by the transmission intensity and variant dominance in categorizing pandemic phases [20, 21]. Epidemiological waves included Wave 1 for the period between February 18, 2020, to May 5, 2020 (circulating ancestral SARS‐CoV‐2); Wave 2 for the period between August 12, 2020, and November 12, 2020 (B1.497 lineage dominant); Wave 3 for the period between November 13, 2020, and July 6, 2021 (Alpha B1.1.7 and Delta B1.620 lineage dominant); Wave 4 for the period between July 7, 2021, and January 29, 2022 (Delta B.1.617); Wave 5 for the period between January 30, 2022, and June 25, 2022 (Omicron BA.1.1 and BA.2.3); Wave 6 for the period between June 26, 2022, and October 16, 2022 (Omicron BA.5); and Wave 7 for the period between October 17, 2022, and later (Omicron BA.5 and BA.2.7). In this study, we included specimen samples collected during Wave 3 to Wave 7, which represent periods with changes in variant dominance to capture the temporal dynamics of SARS‐CoV‐2 during distinct epidemiological phases (Figure 1A,B).

We divided South Korea into six regions including capital, Chungcheong, Gangwon, Gyeongsang, Jeonlla, and Jeju regions [22]. These geographical regions were based on the South Korean map Shapefile, which was produced by the open‐source Database of Global Administrative Areas [23]. We calculated the seropositivity of anti‐S and anti‐N in each region in Korea as mean percentages of positivity of anti‐S and anti‐N and plotted the data using a color gradient from 0 to 100.

To identify the differences in seropositivity of anti‐S and anti‐N by demographic group over time, we used a multivariable Poisson regression model including age, sex, and region, which were based on a priori considerations for the exposure to SARS‐CoV‐2 in Korea [2, 8]. This model produced prevalence ratios (PRs) and corresponding 95% confidence intervals (CIs) to assess the relative likelihood of seropositivity across subgroups [15, 24]. A PR is a measure of association that compares the prevalence of an outcome (i.e., seropositivity) in a specific subgroup to that in a reference group. A PR of 1.0 indicates no significant difference between groups, a PR > 1.0 indicates higher seropositivity in the comparison group relative to the reference, and a PR < 1.0 indicates lower seropositivity. The PR allows for adjustment by covariates such as age, sex, and region, enabling comparison across groups while accounting for potential confounding of the study [25]. We also introduced an interaction term between waves and demographic variables to estimate changes in seropositivity by demographic groups across the Waves. The *p*‐value from the interaction term indicated if there was a significant shift in differences across the waves, for each demographic variable.

We conducted a sensitivity analysis to compare the COVID‐19 vaccination rates (at least one dose) in the population over time using the data weighted against the Korean population Census 2021 for age [26] and nationwide number of COVID‐19 vaccinations from KDCA [27]. The level of statistical significance was considered at *p* < 0.05, and all statistical analyses were performed in R version 4.2.2.

## 3 Results

### 3.1 Demographic Characteristics of Study Participants

A total of 11,504 serum specimens were collected between January 2020 and December 2022 (Table 1). Women accounted for 55.5% (*n* = 6387) of specimens with a range of 54.7% to 56.1% across the study period. By age distribution, most serum specimens were collected from individuals aged 18–44 years (38.1%, *n* = 4388, range of 35.3%–42.8% across the Waves), and the least specimens were collected from children aged 0–17 (7.1% *n* = 822, range 4.3%–8.1%). By geographical distribution, most specimens were collected from the individuals residing in the capital region (48.7%, *n* = 5605, range of 46.3%–52.4%) and the least from individuals residing in the Jeju region (2.0%, *n* = 229, range 1.5%–3.2%). By the number of vaccine doses received, 94.5% of participants had not received any doses in Wave 3; however, vaccination uptake was increased across the Waves, and in Wave 7, 67.0% had received more than three vaccine doses (Table 1 and Figure 1C). The sensitivity analysis revealed no significant differences in the rate of vaccination (at least one dose) between the nationwide dataset (i.e., national vaccination rate reported) and data collected from KHANES (*p* = 0.46, Figure 1D).

**TABLE 1:** Demographic characteristics of study participants tested for SARS‐CoV‐2 antibodies for each cross‐sectional survey in five different epidemic waves between January 2021 and December 2022, South Korea.

Characteristic		Wave 3 (November 13, 2020, and July 6, 2021)	Wave 4 (July 7, 2021, and January 29, 2022)	Wave 5 (January 30, 2022, and June 25, 2022)	Wave 6 (June 26, 2022, and October 16, 2022)	Wave 7 (October 17, 2022, and afterward)	*p*
Sex							0.87
	Male	1478 (45.3%)	1335 (44.1%)	924 (44.27%)	798 (43.9%)	582 (44.5%)	
	Female	1788 (54.7%)	1689(55.9%)	1163(55.7%)	1021 (56.1%)	726 (55.5%)	
Age							< 0.05
	0–17 years	266 (8.1%)	207 (6.8%)	166 (8.0%)	127 (7.0%)	56 (4.3%)	
	18–44 years	1335 (40.9%)	1068 (35.3%)	775 (37.1%)	779 (42.8%)	431 (33.0%)	
	45–64 years	831 (25.4%)	806 (26.7%)	530 (25.4%)	476 (26.2%)	354 (27.1%)	
	≥ 65 years	834 (25.5%)	943 (31.2%)	616 (29.5%)	437 (24.0%)	467 (35.7%)	
COVID‐19 vaccine							< 0.05
	None	3085 (94.5%)	633 (20.9%)	282 (13.5%)	391 (21.5%)	258 (19.7%)	
	1st	124 (3.8%)	461 (15.2%)	11 (0.5%)	10 (0.6%)	8 (0.6%)	
	2nd	57 (1.8%)	1635 (54.1%)	400 (19.2%)	300 (16.5%)	165 (12.6%)	
	3rd	0	295 (9.8%)	1307 (62.6%)	824 (45.3%)	543 (41.5%)	
	≥ 4th	0	0	87 (4.2%)	294 (16.2%)	334 (25.5%)	
Regions							< 0.05
	Capital region	1609(49.3%)	1399(46.3%)	1003(48.1%)	909(50.0%)	685(52.4%)	
	Gangwon region	113(3.5%)	73(2.4%)	93(4.5%)	74(4.1%)	25(1.9%)	
	Chungcheong region	361(11.1%)	379(12.5%)	198(9.5%)	189(10.4%)	174(13.3%)	
	Jeonlla region	342(10.5%)	279(9.2%)	188(9.0%)	183(10.1%)	80(6.1%)	
	Gyeongsang region	791(24.2%)	836(27.6%)	501(24.0%)	409(22.5%)	344(26.3%)	
	Jeju	50(1.5%)	58(1.9%)	66(3.2%)	55(3.0%)	0	
	Not available	0	0	38(1.8%)	0		

### 3.2 Temporal Trends in SARS‐CoV‐2 Antibody Seropositivity

**FIGURE 2:**
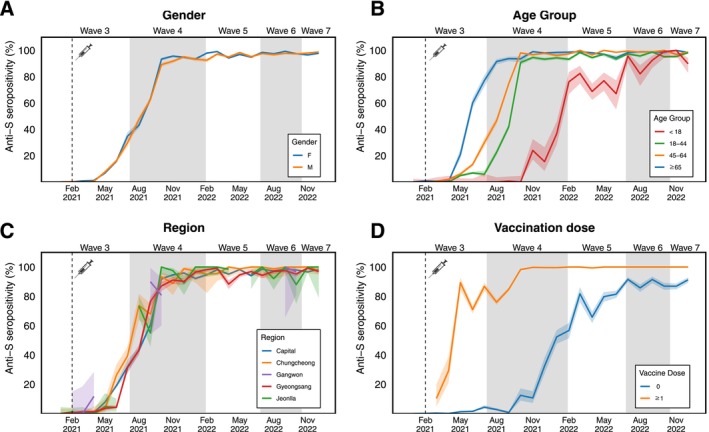
Temporal changes of seropositivity of anti‐S in South Korea, 2021–2022. Seropositivity of anti‐S by demographic characteristic including (A) gender, (B) age, (C) region, and (D) vaccination history. The region was divided into six regions such as the capital, Gangwon, Chungcheong, Jeonlla, Gyeongsang, and Jeju regions in Korea, but Jeju region was not presented here as the limited number of samples in some Waves. We also divided the vaccination status into two categories such as the unvaccinated and any history of vaccination. The grey area surrounding the lines represents the corresponding 95% confidence intervals.

The anti‐S seropositivity rate increased from 7.2% in May 2021 to 98.2% in December 2022 and remained above 93.4% between January 2022 and December 2022 (Figure 1C). No significant sex‐based differences were found in this increase in anti‐S seropositivity across the Waves (Figure 2A, Table 2, and Table S1). However, the increase in anti‐S seropositivity was delayed in younger adults (19–44 years old) during Waves 3–4 and those less than 18 years old compared to the increase in elderly individuals during Waves 3–5 (Table 2 and Figure 2B). For the geographic regions, no significant differences were found in anti‐S seropositivity across the waves (Figure 2C, and Table S1). After adjusting for demographic variables, individuals less than 18 years old had 0.12–0.76 times lower anti‐S seropositivity PR than elderly individuals during Waves 4–5 (Table 2 and Table S1).

**TABLE 2:** Prevalence ratio of anti‐S antibody seropositivity using multi‐variable Poisson regression.

Characteristic	Wave 3 (Nov 13, 2020, to Jul 6, 2021)	Wave 4 (Jul 7, 2021, to Jan 29, 2022)	Wave 5 (Jan 30, 2022, to Jun 25, 2022)	Wave 6 (Jun 26, 2022, and Oct 16, 2022)	Wave 7 (Oct 17, 2022, and afterward)
	PR (95% CI)	PR (95% CI)	PR (95% CI)	PR (95% CI)	PR (95% CI)
Age					
65 + years	Ref	Ref	Ref	Ref	Ref
45–64 years	0.27 (0.24, 0.3)^†^	0.85 (0.82, 0.88)^†^	1.01 (0.96, 1.05)	1 (0.96, 1.05)	0.99 (0.94, 1.04)
18–44 years	0.16 (0.14, 0.18) ^†^	0.71 (0.68, 0.74)^†^	0.98 (0.94, 1.03)	0.98 (0.93, 1.02)	0.98 (0.93, 1.03)
< 18 years	0 (0, Inf)	0.12 (0.10, 0.15)^†^	0.76 (0.70, 0.84)^†^	0.93 (0.84, 1.02)	0.96 (0.84, 1.09)
Sex					
Male	Ref	Ref	Ref	Ref	Ref
Female	0.92 (0.82, 1.02)	1.00 (0.97, 1.03)	1.00 (0.97, 1.03)	1.01 (0.98, 1.04)	0.99 (0.95, 1.03)
Region					
Capital region	Ref	Ref	Ref	Ref	Ref
Gangwon region	0.78 (0.29, 2.09)	0.90 (0.64, 1.27)	1.02 (0.81, 1.3)	0.99 (0.72, 1.36)	1.02 (0.63, 1.64)
Chungcheong region	0.88 (0.66, 1.17)	0.91 (0.84, 0.98)^†^	1.01 (0.94, 1.10)	1.02 (0.93, 1.11)	1.00 (0.91, 1.11)
Jeonlla region	0.21 (0.12, 0.38)^†^	1.05 (0.97, 1.14)	1.02 (0.92, 1.13)	0.98 (0.90, 1.08)	1.01 (0.86, 1.19)
Gyeongsang region	0.30 (0.25, 0.38) ^†^	0.87 (0.84, 0.90)^†^	0.99 (0.95, 1.03)	0.98 (0.94, 1.02)	1.00 (0.95, 1.05)
Jeju	1.85 (0.46, 7.39)	1.19 (0.69, 2.05)	1.02 (0.92, 1.13)	1.02 (0.64, 1.62)	NA

*Note:* A dagger (^†^) indicates a *p*‐value of less than 0.05, which are both considered statistically significant. Capital regions include Seoul, Incheon, and Gyeonggi province. The Chungcheong region includes Daejeon, Sejong, and Chungcheong province. Jeonlla region includes Jeonju, Gwangju, and Jeonlla province. Gyeongsan region includes Daegu, Busan, Ulsan, and Gyeongsang province.

Abbreviations: CI, 95% confidence interval; PR, prevalence ratio; NA, not available.

Regarding vaccination uptake, anti‐S seropositivity reached 93.6% in Wave 4 and remained above this percentage for those who received more than one vaccine dose. However, for those who were not vaccinated, anti‐S seropositivity increased from 0.6% in Wave 3 to 88.0% in Wave 6 (Figure 2D).

The anti‐N seropositivity increased from 0.7% in January 2021 to 2.2% in December 2021 followed by a sharp increase from 1.1% in January 2022 to 68.5% in December 2022 (Figure 1C and Table S2). Sex‐based differences were observed in the anti‐N seropositivity PR (1.06 larger on female compared to the male [95% CI: 1.01–1.10]) (Table S2). After adjusting for demographic variables, individuals younger than 65 years had 1.17–1.83 times higher anti‐N seropositivity PR than elderly individuals during Waves 5–7 (Table 3).

**TABLE 3:** Prevalence ratio of anti‐N antibody seropositivity using multi‐variable Poisson regression;

Characteristic	Wave 3 (Nov 13, 2020, to Jul 6, 2021)	Wave 4 (Jul 7, 2021, to Jan 29, 2022)	Wave 5 (Jan 30, 2022, to Jun 25, 2022)	Wave 6 (Jun 26, 2022, and Oct 16, 2022)	Wave 7 (Oct 17, 2022, and afterward)
	PR (95% CI)	PR (95% CI)	PR (95% CI)	PR (95% CI)	PR (95% CI)
Age					
65 + years	Ref	Ref	Ref	Ref	Ref
45–64 years	1.59 (0.82, 3.08)	1.01 (0.73, 1.39)	1.27 (1.06, 1.39)^†^	1.25 (1.17, 1.34)^†^	1.17 (1.1, 1.25)^†^
18–44 years	1.22 (0.62, 2.40)	0.77 (0.55, 1.08)	1.64 (1.50, 1.78)^†^	1.32 (1.23, 1.41)^†^	1.24 (1.16, 1.32)^†^
< 18 years	0 (0, Inf) *	0.79 (0.36, 1.74)	1.83 (1.60, 2.11)^†^	1.78 (1.59, 2.00) ^†^	1.6 (1.39, 1.84)^†^
Sex					
Male	Ref	Ref	Ref	Ref	Ref
Female	1.33 (0.88, 2.01)	1.04 (0.83, 1.31)	1.12 (1.06, 1.18)^†^	1.07 (1.03, 1.12) ^†^	1.06 (1.1, 1.25)^†^
Region					
Capital region†	Ref	Ref	Ref	Ref	Ref
Gangwon region	3.54 (0.49, 25.54)	1.49 (0.21, 10.65)	0.68 (0.39, 1.17)	0.88 (0.57, 1.36)	0.99 (0.55, 1.78)
Chungcheong region	0.64 (0.16, 2.62)	0.8 (0.44, 1.46)	0.91 (0.78, 1.05)	0.95 (0.84, 1.07)	1.02 (0.91, 1.15)
Jeonlla region	0 (0, Inf)	0 (0.03, inf)	0.66 (0.54, 0.82)^†^	0.92 (0.81, 1.05)	0.83 (0.67, 1.04)
Gyeongsang region	1.19 (0.73, 1.96)	0.30 (0.19, 0.47)^†^	0.89 (0.83, 0.95)^†^	0.95 (0.90, 1.01)	1.00 (0.94, 1.06)
Jeju	0 (0, Inf)	0 (0, Inf)	0.15 (0.02, 1.08)	0.92 (0.81, 1.05)	NA

*Note:* A dagger (^†^) indicates a *p*‐value of less than 0.05, which was considered statistically significant. Capital regions include Seoul, Incheon, and Gyeonggi province. The Chungcheong region includes Daejeon, Sejong, and Chungcheong province. Jeonlla region includes Jeonju, Gwangju, and Jeonlla province. Gyeongsan region includes Daegu, Busan, Ulsan, and Gyeongsang province.

Abbreviations: CI, 95% confidence interval; PR, prevalence ratio; NA, not available.

### 3.3 Changes in the Geographic Distribution of SARS‐CoV‐2 Antibody Seropositivity

**FIGURE 3:**
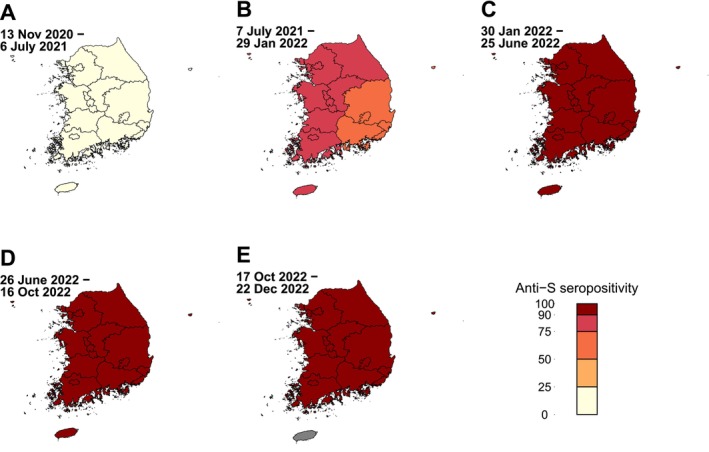
Geographical distribution of SARS‐CoV‐2 spike antibody positivity in South Korea during January 2021 and December 2022. The seropositivity of anti‐S was determined for six provincial regions including the capital, Chungcheong, Gangwon, Gyeongsang, Jeonlla, and Jeju regions during the five SARS‐CoV‐2 epidemiological waves. The epidemiological waves included (A) Wave 3 for November 13, 2020, and July 6, 2021; (B) Wave 4 for July 7, 2021, and January 29, 2022; (C) Wave 5 for January 30, 2022, and June 25, 2022; (D) Wave 6 for June 26, 2022, and October 16, 2022; and (E) Wave 7 for October 17, 2022, and later. The color gradient indicates the range of seropositivity, and areas with no specimens collected are shaded gray (i.e., Jeju region).

**FIGURE 4:**
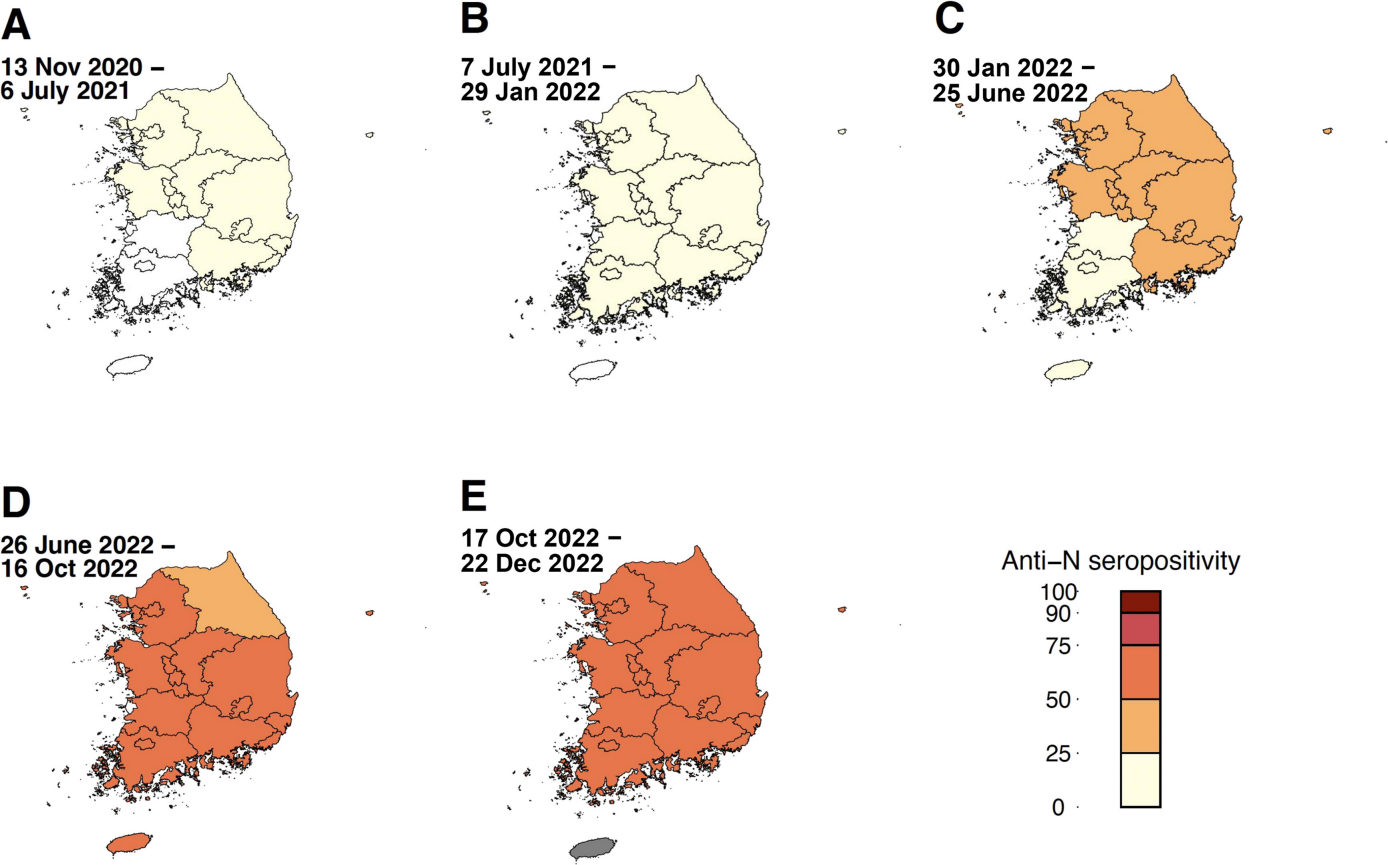
Geographical distribution of SARS‐CoV‐2 nucleocapsid antibody positivity in South Korea during January 2021 and December 2022. The seropositivity of anti‐S was determined for six provincial regions including the capital, Chungcheong, Gangwon, Gyeongsang, Jeonlla, and Jeju regions during the five SARS‐CoV‐2 epidemiological waves. The epidemiological waves included (A) Wave 3 for November 13, 2020, and July 6, 2021; (B) Wave 4 for July 7, 2021, and January 29, 2022; (C) Wave 5 for January 30, 2022, and June 25, 2022; (D) Wave 6 for June 26, 2022, and October 16, 2022; and (E) Wave 7 for October 17, 2022, and later. The color gradient indicates the range of seropositivity, and areas with no specimens collected are shaded gray (i.e., Jeju region).

After adjusting for demographic variables, Gyeongsang region had 0.30–0.87 times lower anti‐S seropositivity PR than the capital region in Wave 3‐4 (Figure 3, Table 2, and Table S1). The anti‐N seropositivity PR was 0.30–0.89 times lower in Gyeongsang region in Waves 4–5 and 0.66 times lower in Jeonlla region in Wave 5 (Figure 4, Table 3, and Table S2).

## 4 Discussion

The COVID‐19 pandemic has heavily burdened healthcare systems globally. Disparities in susceptibility (i.e., immunity level caused by the COVID‐19 vaccination and infection) of individuals based on their population demographics have been reported in many countries [28, 29]. Identifying and addressing the disparity at the country level is critical to ensure preparedness against the next pandemic and developing effective response strategies including effective utilization of public health resources.

### 4.1 Temporal Trends in SARS‐CoV‐2 Antibody Seropositivity

We identified that the overall anti‐S seropositivity rapidly increased between May and October 2021 after the COVID‐19 vaccination was introduced in February 2021. However, the anti‐S seropositivity PR was lower among children than in the elderly population during Waves 4–5. This is likely because the COVID‐19 vaccination was introduced at different times for different age groups in Korea; that is, the vaccine was introduced first for individuals who were 65 years or older and later on for the age group of 18 years and younger [30]. The finding for the delayed increase in the anti‐S among children was also observed in other countries, as the vaccine recommendation for the children was introduced later compared to the older adults [10, 31, 32].

After the Omicron variant was first introduced in Korea on November 25, 2021 [8], the number of new daily cases reached new heights since the pandemic began [7]. In this study, we also identified an increase in the anti‐N seroprevalence from Wave 4 onward. This is likely due to the increased number of Omicron infection cases. Furthermore, due to the antigenic exposures (i.e., COVID‐19 vaccination and SARS‐CoV‐2 breakthrough infections), the anti‐S seroprevalence, which crossed 90% by January 2022, was maintained above until December 2022. These findings of increased anti‐S and anti‐N seroprevalence have been observed in other regions in other countries [9, 33, 34]. This finding is corroborated by the global seroprevalence study, which demonstrated that the infection‐induced seroprevalence increased to 48% in Europe and 34% in America after the emergence of Omicron [12]. Furthermore, we noticed a higher anti‐N seropositivity PR in individuals younger than 65 years than in elderly (≥ 65 years) individuals. This is likely because public health communication against SARS‐CoV‐2 infection emphasized that the SARS‐CoV‐2 infection for the elderly was more clinically critical than other age groups. This finding aligns with the findings of other studies suggesting that elderly people are more vulnerable to the infection and were the main target population group regarding messaging about the importance of vaccination, booster doses, and strict adherence to public health measures. These insights point toward the need for age‐specific communication strategies against SARS‐CoV‐2 infection [4, 35, 36].

### 4.2 Changes in the Geographic Distribution of SARS‐CoV‐2 Antibody Seropositivity

The anti‐S seropositivity PR in Wave 3 was lower in the Gyeongsang region than in the capital region, aligning with the lower COVID‐19 vaccination rate in the elderly population in the Gyeongsang region than in the capital region (Figure S1). However, after Wave 3, there was no disparity in the anti‐S seropositivity PR across the regions. This is likely affected by the comprehensive government strategy to improve the nationwide vaccination uptake, which included ensuring reliable vaccine supply and storage, prioritizing efficient administration, and being transparent with the public about adverse reactions to the vaccine [37]. Implementing such targeted and adaptive policies would be critical not only for improving population‐level immunity but also for reducing the risk of transmission and severe clinical outcomes.

The anti‐N seropositivity PR was lower in Gyeongsang and Jeju regions than in the capital region in Waves 4 and 5, respectively. The Omicron variant was first identified in the capital region, and superspreading events were then identified in the capital region, which spread to nearby regions including Chungcheong, Jeonlla, and Gangwon regions in Korea [8]. Because Gyeongsang and Jeju regions are located far from the capital region compared to the other regions, the anti‐N seropositivity PR in these regions was probably lower.

Sero‐surveillance of SARS‐CoV‐2 is expected to reveal seropositivity trends and capture undetected or unreported infections that are not included in case‐based surveillance systems of SARS‐CoV‐2 [9, 38, 39]. To monitor the SARS‐CoV‐2 immune level in the community and to identify occupational risks, sero‐surveillance of SARS‐COV‐2 has been implemented in many countries in response to the pandemic [9, 40]. However, community‐based sero‐surveillance has serval limitations. First, participation may skew toward individuals with higher health awareness or those more concerned about COVID‐19 [4]. Second, waning kinetics of antibodies may vary over time, making it crucial to identify the exact timing of sample collection relative to infection or vaccination [12]. Third, large‐scale sero‐surveillance requires abundant resources to be sustainably conducted in the long run [41]. Therefore, the existing nationwide health survey framework, as was used in the present study, can be a low‐cost alternative to leverage existing laboratory surveillance infrastructure to generate seroprevalence data [9]. Therefore, the key strength of this study is that it is the first population‐based study conducted over two consecutive years to capture nationwide changes in seroprevalence after the introduction of COVID‐19 vaccination and the Omicron variant. Furthermore, we used the Korean national SARS‐CoV‐2 infection notification database, which included data from the extended community COVID‐19 screening centers that operated during the study period and the national immunization database. However, our study has some limitations. First, the study population might have been biased because individuals who agreed to participate in the survey for sample collection were likely to be more health conscious. Second, individual immune responses are bound to be influenced by age, comorbidities, severity of infection, and vaccination type (Figure S2). This variability can lead to challenges in accurately interpreting seroprevalence among both vaccinated and previously infected individuals [9]. Furthermore, we could not determine the true levels of exposure or immunity because of the waning kinetics of anti‐S and anti‐N antibodies and varying patterns of exposure to infection or vaccination across different geographic regions (Figures S2 and S3). Third, antibody levels do not directly equate to immune levels in individuals, as antibody levels needed for protection vary and do not capture T‐cell responses [41]. However, data based on electrochemiluminescence immunoassay, which we conducted in this study, strongly and positively correlate with results from the neuralization assay [42, 43], which has been widely used globally in sero‐surveillance studies [11, 12, 33].

In conclusion, leveraging an existing Korean nationwide survey system, we observed an increase in SARS‐CoV‐2 antibody levels in line with epidemiological data on SARS‐CoV‐2 and the rollout of the COVID‐19 vaccine. Furthermore, we identified that almost all of the study population achieved COVID‐19 seropositivity in early 2022. Additional research is needed to finetune sero‐surveillance data leveraging an existing nationwide health survey system to prepare for a future pandemic in the country.

## Author Contributions

Conceptualization: J.L., and S.R.; methodology: A. K, J.L., and S.R.; validation: H.N.D., E.Y.J., H.J., G.R., K.O., S.L.K., J.L., and S.R.; formal analysis: A. K, C.A., H.N.D., E.Y.J., Y.N. and S.C.; investigation: A. K, C.A., H.N.D., E.Y.J., Y.N., S.C., H.S.J., and J.L.; resources: J.L.; data curation: A. K, C.A., H.N.D., E.Y.J., Y.N. and S.C.; writing – original draft preparation: A.K., C.A., H.N.D., E.Y.J., Y.N., S.C., J.L., and S.R.; writing – review and editing: S.C., and S.R.; visualization: S.C., T.K., and S.L.; supervision: S.L., and S.R. All authors have read and agreed to the published version of the manuscript.

## Conflicts of Interest

The authors declare no conflicts of interest.

## Disclaimer

The funding bodies had no role in study design, data collection and analysis, preparation of the manuscript, or the decision to publish.

## Supporting information


**Table S1.** Full parameter estimates from a multivariable Poisson regression of anti‐S seropoitivity. *Note*: CI, confidence interval; PR, prevalence ratio. Capital regions include Seoul, Incheon, and Gyeonggi province. The Chungcheong region includes Daejeon, Sejong, and Chungcheong province. Jeonlla region includes Jeonju, Gwangju, and Jeonlla province. Gyeongsang region includes Daegu, Busan, Ulsan, and Gyeongsang province. NA indicates not available.
**Table S2.** Full parameter estimates from a multivariable Poisson regression of anti‐N seropositivity. *Note*: CI, confidence interval; PR, prevalence ratio. Capital regions include Seoul, Incheon, and Gyeonggi province. The Chungcheong region includes Daejeon, Sejong, and Chungcheong province. Jeonlla region includes Jeonju, Gwangju, and Jeonlla province. Gyeongsang region includes Daegu, Busan, Ulsan, and Gyeongsang province. NA indicates not available.
**Figure S1.** Cumulative rate of COVID‐19 vaccination in different regions in South Korea during epidemic Wave 3 (November 13, 2020, and July 6, 2021). The boxplot indicates the COVID‐19 vaccination rate in the regions during epidemic Wave 3. The capital region includes Seoul, Incheon, and Gyeonggi province. The Chungcheong region includes Daejeon, Sejong, and Chungcheong province. Jeonlla region includes Jeonju, Gwangju, and Jeonlla province. Gyeongsan region includes Daegu, Busan, Ulsan, and Gyeongsang province.
**Figure S2.** Temporal kinetics of SARS‐CoV‐2 spike antibody for individuals with no infection history after receiving the second dose of different types of vaccination. The vaccination type includes mRNA vaccine from Pfizer‐BioNTech (A), Moderna COVID‐19 vaccine (B), and viral vector‐based vaccine from Oxford–AstraZeneca (C). The box plot indicates the titer of spike antibodies by sampling time, which was based on the day between the last day of vaccination and the day the specimen was collected.
**Figure S3.** Temporal kinetics of SARS‐CoV‐2 nucleocapsid antibody for individuals with a history of infection and spike antibody for individuals with an infection history without having COVID‐19 vaccination. The sampling time is based on the day between the notification of infection and the day the specimen was collected. Dots represent titers of nucleocapsid antibody (A) and spike antibody (B). The thick curve indicates the trend using GAM with the 95% confidence interval (shaded area).

## Data Availability

The data that support the findings of this study are available on reasonable request to the corresponding author. The data are not publicly available due to privacy or ethical restrictions.
