# Supplementary material for: Seroprevalence Trends of Antibodies to SARS‐CoV‐2 in South Korea, 2021–2022: A Repeated Cross‐Sectional Study
Source: Influenza Other Respir Viruses. 2025 Jun 3;19(6):e70117. doi: 10.1111/irv.70117 (PMC12134085; doi:10.1111/irv.70117)
Supplement: Supplementary file 1 — Table S1. Full parameter estimates from a multivariable Poisson regression of anti‐S seropoitivity. Note: CI, confidence interval; PR, prevalence ratio. Capital regions include Seoul, Incheon, and Gyeonggi province. The Chungcheong region includes Daejeon, Sejong, and Chungcheong province. Jeonlla region includes Jeonju, Gwangju, and Jeonlla province. Gyeongsang region includes Daegu, Busan, Ulsan, and Gyeongsang province. NA indicates not available. Table S2. Full parameter estimates from a multivariable Poisson regression of anti‐N seropositivity. Note: CI, confidence interval; PR, prevalence ratio. Capital regions include Seoul, Incheon, and Gyeonggi province. The Chungcheong region includes Daejeon, Sejong, and Chungcheong province. Jeonlla region includes Jeonju, Gwangju, and Jeonlla province. Gyeongsang region includes Daegu, Busan, Ulsan, and Gyeongsang province. NA indicates not available. Figure S1. Cumulative rate of COVID‐19 vaccination in different regions in South Korea during epidemic Wave 3 (November 13, 2020, and July 6, 2021). The boxplot indicates the COVID‐19 vaccination rate in the regions during epidemic Wave 3. The capital region includes Seoul, Incheon, and Gyeonggi province. The Chungcheong region includes Daejeon, Sejong, and Chungcheong province. Jeonlla region includes Jeonju, Gwangju, and Jeonlla province. Gyeongsan region includes Daegu, Busan, Ulsan, and Gyeongsang province. Figure S2. Temporal kinetics of SARS‐CoV‐2 spike antibody for individuals with no infection history after receiving the second dose of different types of vaccination. The vaccination type includes mRNA vaccine from Pfizer‐BioNTech (A), Moderna COVID‐19 vaccine (B), and viral vector‐based vaccine from Oxford–AstraZeneca (C). The box plot indicates the titer of spike antibodies by sampling time, which was based on the day between the last day of vaccination and the day the specimen was collected. Figure S3. Temporal kinetics of SARS‐CoV‐2 nucleocapsid antibody for ind [file IRV-19-e70117-s001.docx]

**Supplementary Materials**

**Contents**

Supplementary Tables

Supplementary Figures

**Supplementary Table 1.** Full parameter estimates from a multivariable Poisson regression of anti-S seropositivity.

| **Characteristic** | **PR (95% CI)** | ***P*-value for parameter estimate (two-tailed Z test)** |
| --- | --- | --- |
| Wave |  |  |
| Wave 7 | Reference |  |
| Wave 6 | 0.99 (0.92, 1.06) | 0.7 |
| Wave 5 | 0.98 (0.92, 1.05) | 0.53 |
| Wave 4 | 0.98 (0.92, 1.04) | 0.45 |
| Wave 3 | 0.18 (0.16, 0.2) | <0.01 |
| Sex |  |  |
| Male | Reference |  |
| Female | 0.99 (0.95, 1.03) | 0.56 |
| Age |  |  |
| 65+ years | Reference |  |
| 45-64 years | 0.99 (0.94, 1.04) | 0.68 |
| 18-44 years | 0.98 (0.93, 1.03) | 0.35 |
| <18 years | 0.96 (0.84, 1.09) | 0.52 |
| Region |  |  |
| Capital region | Reference |  |
| Gangwon region | 1.02 (0.63, 1.64) | 0.93 |
| Chungcheong region | 1 (0.91, 1.11) | 0.92 |
| Jeonlla region | 1.01 (0.86, 1.19) | 0.86 |
| Gyeongsang region | 1 (0.95, 1.05) | 0.94 |
| Jeju region | 1.85 (0.46, 7.39) | 0.39 |
| Wave*Sex |  |  |
| Wave 7*Male | Reference |  |
| Wave 6*Female | 1.02 (0.97, 1.07) | 0.37 |
| Wave 5*Female | 1.01 (0.96, 1.06) | 0.69 |
| Wave 4*Female | 1.01 (0.97, 1.06) | 0.61 |
| Wave 3*Female | 0.93 (0.82, 1.04) | 0.19 |
| Wave*Age |  |  |
| Wave 7*Age≥65 | Reference |  |
| Wave 6*Age45-64 | 1.01 (0.95, 1.09) | 0.68 |
| Wave 5*Age45-64 | 1.02 (0.95, 1.09) | 0.58 |
| Wave 4*Age45-64 | 0.86 (0.81, 0.92) | <0.01 |
| Wave 3*Age45-64 | 0.27 (0.24, 0.31) | <0.01 |
| Wave 6*Age18-44 | 1 (0.93, 1.07) | 0.98 |
| Wave 5*Age18-44 | 1.01 (0.94, 1.08) | 0.87 |
| Wave 4*Age18-44 | 0.73 (0.68, 0.78) | <0.01 |
| Wave 3*Age18-44 | 0.16 (0.14, 0.19) | <0.01 |
| Wave 6*Age<18 | 0.97 (0.82, 1.14) | 0.71 |
| Wave 5*Age<18 | 0.8 (0.68, 0.94) | 0.01 |
| Wave 4*Age<18 | 0.13 (0.1, 0.16) | <0.01 |
| Wave 3*Age<18 | 0 (0, Infinite) | 0.89 |
| Wave*Region |  |  |
| Wave 7*Capital region | Reference |  |
| Wave 6*Gangwon region | 0.97 (0.55, 1.72) | 0.92 |
| Wave 5*Gangwon region | 1 (0.59, 1.71) | 0.99 |
| Wave 4*Gangwon region | 0.88 (0.49, 1.58) | 0.67 |
| Wave 3*Gangwon region | 0.76 (0.26, 2.28) | 0.63 |
| Wave 6*Chungcheong region | 1.01 (0.89, 1.15) | 0.85 |
| Wave 5*Chungcheong region | 1.01 (0.89, 1.15) | 0.88 |
| Wave 4*Chungcheong region | 0.9 (0.8, 1.02) | 0.1 |
| Wave 3*Chungcheong region | 0.88 (0.65, 1.18) | 0.39 |
| Wave 6*Jeonlla region | 0.97 (0.81, 1.17) | 0.75 |
| Wave 5*Jeonlla region | 1.01 (0.83, 1.21) | 0.95 |
| Wave 4*Jeonlla region | 1.03 (0.86, 1.24) | 0.71 |
| Wave 3*Jeonlla region | 0.21 (0.11, 0.38) | <0.01 |
| Wave 6*Gyeongsang region | 0.98 (0.92, 1.05) | 0.55 |
| Wave 5*Gyeongsang region | 0.99 (0.93, 1.05) | 0.75 |
| Wave 4*Gyeongsang region | 0.87 (0.82, 0.93) | <0.01 |
| Wave 3*Gyeongsang region | 0.31 (0.25, 0.38) | <0.01 |
| Wave 6*Jeju region | 0.55 (0.13, 2.39) | 0.43 |
| Wave 5*Jeju region | 0.55 (0.13, 2.37) | 0.43 |
| Wave 4*Jeju region | 0.64 (0.15, 2.86) | 0.56 |

Note: PR, prevalence ratio; CI, confidence interval. Capital regions include Seoul, Incheon, and Gyeonggi province. The Chungcheong region includes Daejeon, Sejong, and Chungcheong province. Jeonlla region includes Jeonju, Gwangju, and Jeonlla province. Gyeongsang region includes Daegu, Busan, Ulsan, and Gyeongsang province. NA indicates not available.

**Supplementary Table 2.** Full parameter estimates from a multivariable Poisson regression of anti-N seropositivity.

| **Characteristic** | **PR (95% CI)** | ***P*-value for parameter estimate (two-tailed Z test)** |
| --- | --- | --- |
| Wave |  |  |
| Wave 7 | Reference |  |
| Wave 6 | 0.78 (0.71, 0.86) | <0.01 |
| Wave 5 | 0.41 (0.37, 0.46) | <0.01 |
| Wave 4 | 0.03 (0.02, 0.04) | <0.01 |
| Wave 3 | 0 (0, 0.01) | <0.01 |
| Sex |  |  |
| Male | Reference |  |
| Female | 1.06 (1.01, 1.1) | 0.02 |
| Age |  |  |
| 65+ years | Reference |  |
| 45-64 years | 1.17 (1.1, 1.25) | <0.01 |
| 18-44 years | 1.24 (1.16, 1.32) | <0.01 |
| <18 years | 1.6 (1.39, 1.84) | <0.01 |
| Region |  |  |
| Capital region | Reference |  |
| Gangwon region | 0.99 (0.55, 1.78) | 0.97 |
| Chungcheong region | 1.02 (0.91, 1.15) | 0.73 |
| Jeonlla region | 0.83 (0.67, 1.04) | 0.11 |
| Gyeongsang region | 1 (0.94, 1.06) | 0.95 |
| Jeju | NA |  |
| Wave*Sex |  |  |
| Wave 7*Male | Reference |  |
| Wave 6*Female | 1.01 (0.96, 1.08) | 0.63 |
| Wave 5*Female | 1.06 (0.99, 1.13) | 0.11 |
| Wave 4*Female | 0.99 (0.78, 1.25) | 0.9 |
| Wave 3*Female | 1.26 (0.83, 1.91) | 0.28 |
| Wave*Age |  |  |
| Wave 7*age≥65 | Reference |  |
| Wave 6*Age45-64 | 1.07 (0.97, 1.17) | 0.17 |
| Wave 5*Age45-64 | 1.09 (0.97, 1.21) | 0.14 |
| Wave 4*Age45-64 | 0.86 (0.62, 1.19) | 0.37 |
| Wave 3*Age45-64 | 1.36 (0.7, 2.63) | 0.36 |
| Wave 6*Age18-44 | 1.07 (0.97, 1.17) | 0.17 |
| Wave 5*Age18-44 | 1.32 (1.19, 1.47) | <0.01 |
| Wave 4*Age18-44 | 0.62 (0.44, 0.88) | 0.01 |
| Wave 3*Age18-44 | 0.99 (0.5, 1.95) | 0.97 |
| Wave 6*Age<18 | 1.11 (0.93, 1.33) | 0.24 |
| Wave 5*Age<18 | 1.15 (0.94, 1.39) | 0.17 |
| Wave 4*Age<18 | 0.49 (0.22, 1.1) | 0.08 |
| Wave 3*Age<18 | NA |  |
| Wave*Region |  |  |
| Wave 7*Capital region | Reference |  |
| Wave 6*Gangwon region | 0.89 (0.43, 1.86) | 0.75 |
| Wave 5*Gangwon region | 0.68 (0.31, 1.53) | 0.36 |
| Wave 4*Gangwon region | 1.51 (0.19, 11.77) | 0.69 |
| Wave 3*Gangwon region | 3.59 (0.46, 28.21) | 0.22 |
| Wave 6*Chungcheong region | 0.93 (0.78, 1.1) | 0.39 |
| Wave 5*Chungcheong region | 0.89 (0.74, 1.07) | 0.21 |
| Wave 4*Chungcheong region | 0.78 (0.42, 1.45) | 0.43 |
| Wave 3*Chungcheong region | 0.63 (0.15, 2.58) | 0.52 |
| Wave 6*Jeonlla region | 1.11 (0.86, 1.43) | 0.44 |
| Wave 5*Jeonlla region | 0.8 (0.59, 1.08) | 0.15 |
| Wave 4*Jeonlla region | 0 (0, Inf) | 1 |
| Wave 3*Jeonlla region | 0 (0, Inf) | 1 |
| Wave 6*Gyeongsan region | 0.95 (0.87, 1.03) | 0.22 |
| Wave 5*Gyeongsan region | 0.89 (0.81, 0.97) | 0.01 |
| Wave 4*Gyeongsan region | 0.3 (0.19, 0.47) | <0.01 |
| Wave 3*Gyeongsan region | 1.19 (0.72, 1.96) | 0.49 |
| Wave 6*Jeju region | Inf (0, Inf) | 0.98 |
| Wave 5*Jeju region | Inf (0, Inf) | 0.98 |
| Wave 4*Jeju region | Inf (0, Inf) | 0.98 |

Note: PR, prevalence ratio; CI, confidence interval. Capital regions include Seoul, Incheon, and Gyeonggi province. The Chungcheong region includes Daejeon, Sejong, and Chungcheong province. Jeonlla region includes Jeonju, Gwangju, and Jeonlla province. Gyeongsang region includes Daegu, Busan, Ulsan, and Gyeongsang province. NA indicates not available.


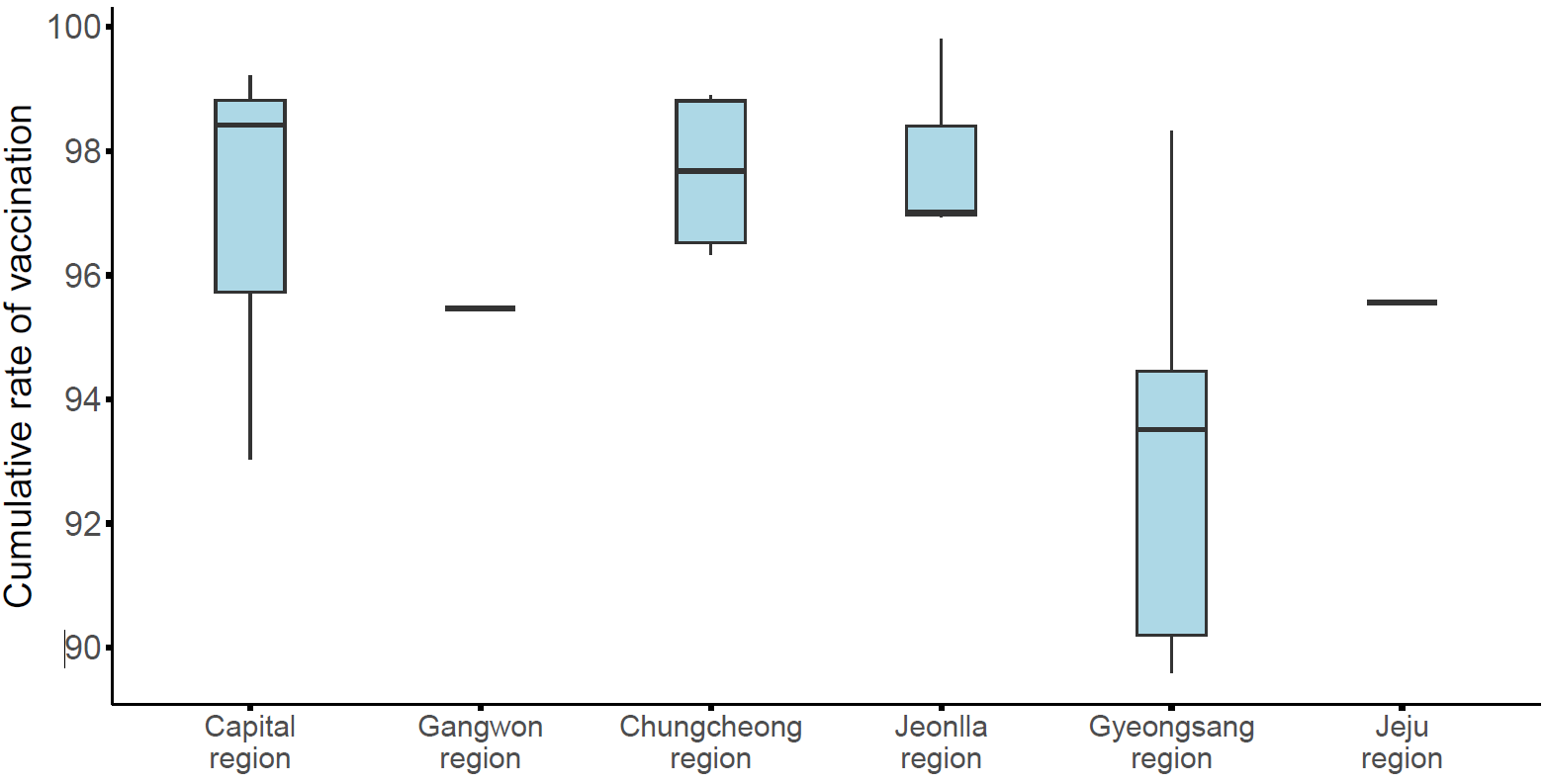


**Supplementary Figure 1.** Cumulative rate of COVID-19 vaccination in different regions in South Korea during epidemic Wave 3 (13 November 2020 and 6 July 2021). The boxplot indicates the COVID-19 vaccination rate in the regions during epidemic Wave 3. The capital region includes Seoul, Incheon, and Gyeonggi province. The Chungcheong region includes Daejeon, Sejong, and Chungcheong province. Jeonlla region includes Jeonju, Gwangju, and Jeonlla province. Gyeongsan region includes Daegu, Busan, Ulsan, and Gyeongsang province.

**
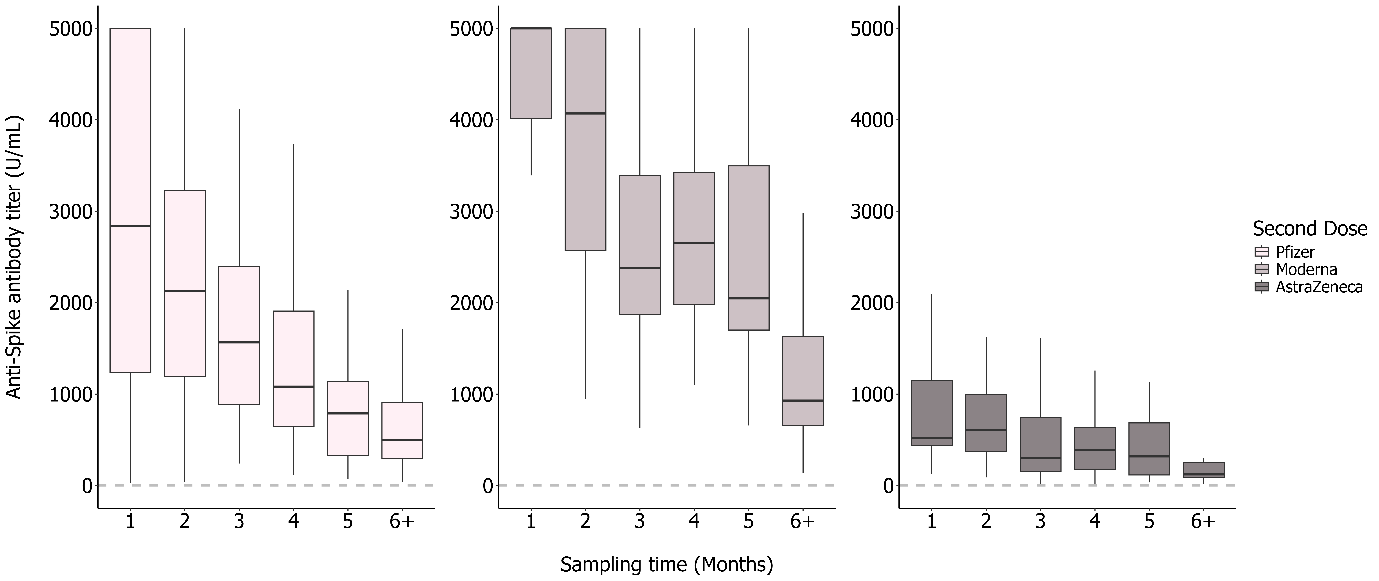
**

**Supplementary Figure 2.** Temporal kinetics of SARS-CoV-2 spike antibody for individuals with no infection history after receiving the second dose of different types of vaccination. The vaccination type includes mRNA vaccine from Pfizer-BioNTech (A), Moderna COVID-19 vaccine (B), and viral vector-based vaccine from Oxford–AstraZeneca (C). The box plot indicates the titer of spike antibodies by sampling time, which was based on the day between the last day of vaccination and the day the specimen was collected.


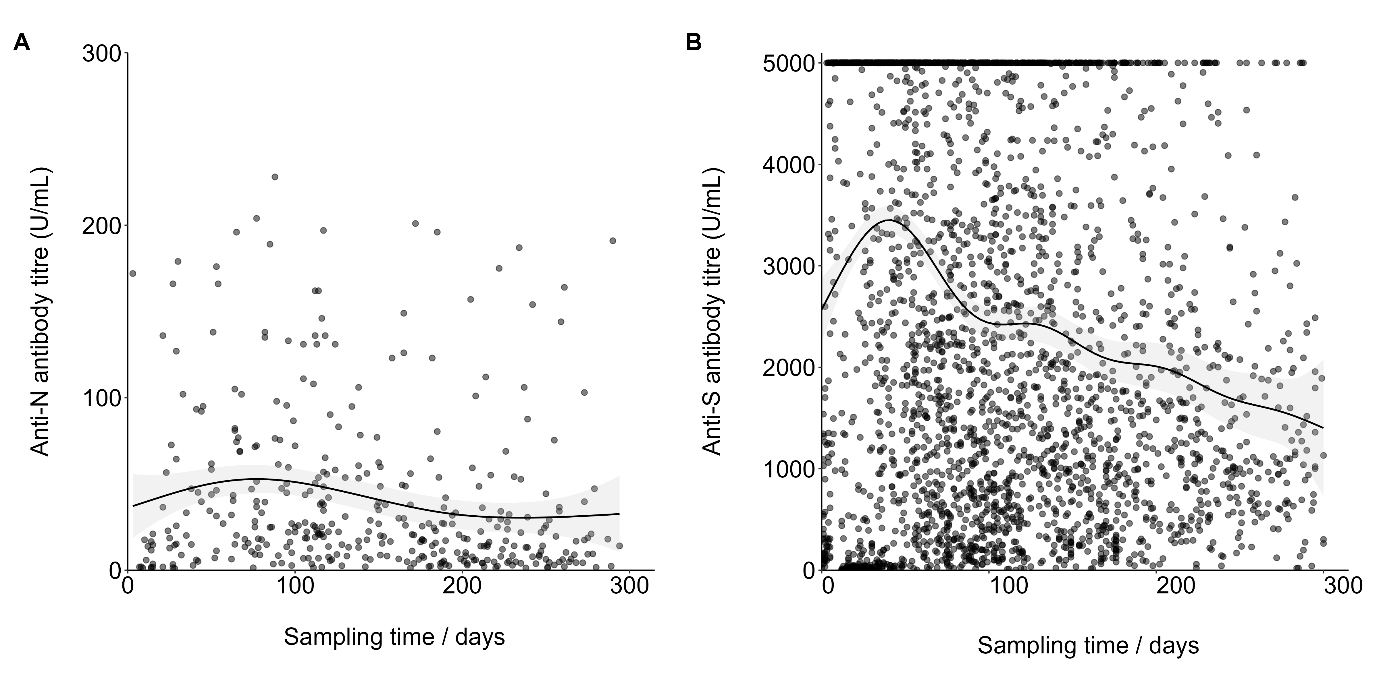


**Supplementary Figure 3.** Temporal kinetics of SARS-CoV-2 nucleocapsid antibody for individuals with a history of infection and spike antibody for individuals with an infection history without having COVID-19 vaccination. The sampling time is based on the day between the notification of infection and the day the specimen was collected. Dots represent titers of nucleocapsid antibody (A) and spike antibody (B). The thick curve indicates the trend using GAM with the 95% confidence interval (shaded area).
